# Syncope as the Initial Manifestation of a Late-Stage Renal Cell Carcinoma With Metastasis to the Brain

**DOI:** 10.7759/cureus.16185

**Published:** 2021-07-05

**Authors:** Grace W Ying, Hafiz Muhammad Jeelani, Michael J Chaney, Xuanzhen Piao, Nikita Jain, Maryna Shayuk, Om Parkash

**Affiliations:** 1 Internal Medicine, Chicago Medical School, Northwestern Medicine McHenry Hospital, McHenry, USA; 2 Medicine, Chicago Medical School, Rosalind Franklin University of Medicine and Science, North Chicago, USA; 3 Internal Medicine, Chicago Medical School, Rosalind Franklin University of Medicine and Science, North Chicago, USA

**Keywords:** renal cell carcinoma, brain metastasis, cancer, large tumor, craniotomy, computed tomography, primary care

## Abstract

Renal cell carcinoma (RCC) is the most common neoplasm that arises from renal parenchyma. About one-third of patients with RCC develop metastatic spread, with common sites including the lung, liver, bone, adrenal gland, and brain. Distant metastases can be difficult to detect unless symptoms appear. We report a case of a 56-year-old female who presented to the emergency department with the unresponsiveness of unknown duration. She underwent a thorough laboratory workup, and the computed tomography (CT) scan revealed a retroperitoneal mass originating from the right kidney and a large hemorrhagic brain mass in the left frontal lobe. The patient underwent emergent full craniotomy for tumor removal, and histology confirmed metastatic RCC. Since several patients with RCC are asymptomatic, the slow growth of tumors leading to distant metastasis can be overlooked. Thus, this case demonstrates the importance of early detection of RCC to help prevent or delay further disease progression.

## Introduction

Renal cell carcinoma (RCC) accounts for about 5% of cancer in adult men and 3% in adult women; it is the most common primary renal neoplasm accounting for about 90% [1-2]. Risk factors include male gender, age over 50 years, smoking, heavy metal exposure, end-stage renal disease, and von Hippel-Lindau syndrome [3]. The classical presentation of RCC includes hematuria, flank pain, and abdominal mass. Less commonly, it can be associated with inferior vena cava (IVC) thrombosis and compression of surrounding structures like the bowel and ureters. An estimated 18% of cases of RCC are metastatic [2], with a five-year cumulative incidence of brain metastasis from RCC found to be 9.8% [4]. Factors such as primary tumor size and location are integral in whether or not a metastasis produces mass effect symptoms or remains clinically silent and, therefore, undetected. The exact rate at which an RCC brain metastasis will cause symptoms is hard to predict. In this case report, we present a case of RCC metastasis to the brain affecting the mental status and the subsequent surgical decompression and medical management.

## Case presentation

**Figure 1:**
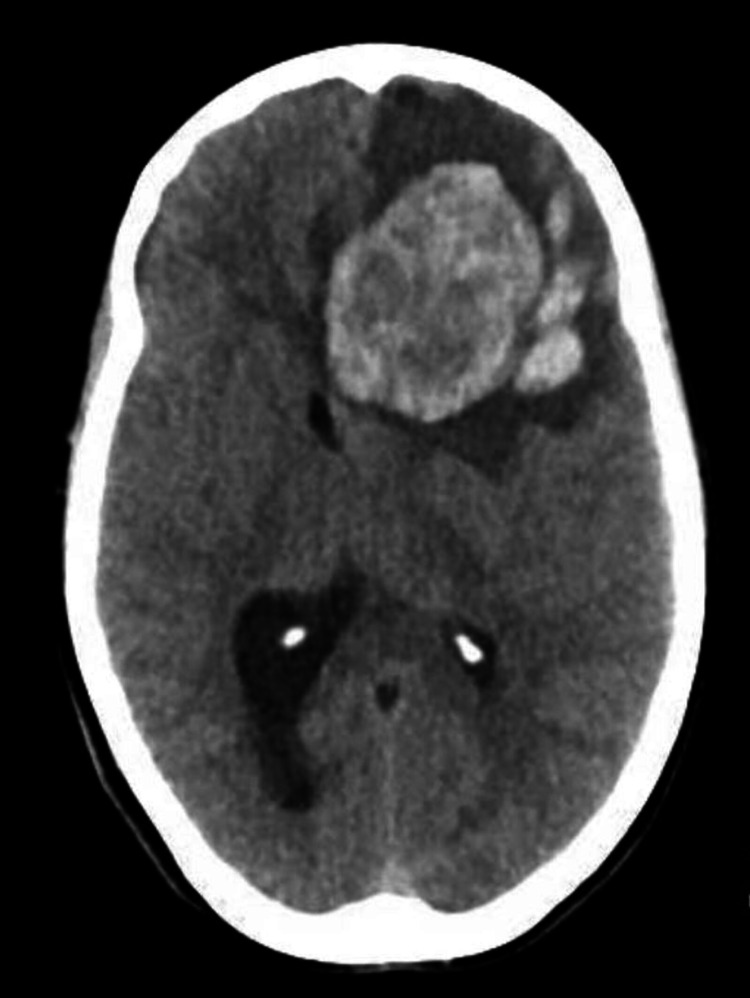
Noncontrast head CT in axial plane preceding surgical decompression showing a large left frontal lobe mass measuring 4.5 x 5.2 cm with anterior and inferior acute intraparenchymal hemorrhage and significant surrounding edema and mass effect.

**Figure 2:**
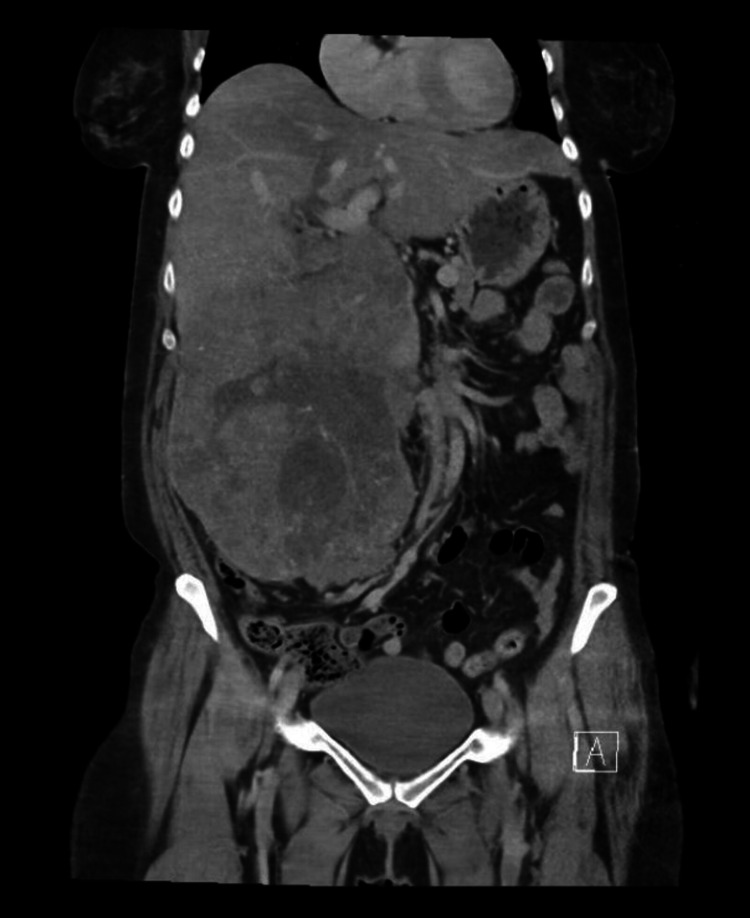
Abdominal CT with contrast in coronal plane showing a very large tumor in right mid-to-lower abdomen arise from the right kidney, which is being totally replaced. The mass is at least 20.1 x 15.6 x 23.7 cm with tumor thrombus extending into the inferior vena cava. Grossly there is no hepatomegaly, ascites, or metastasis in the liver.

**Figure 3:**
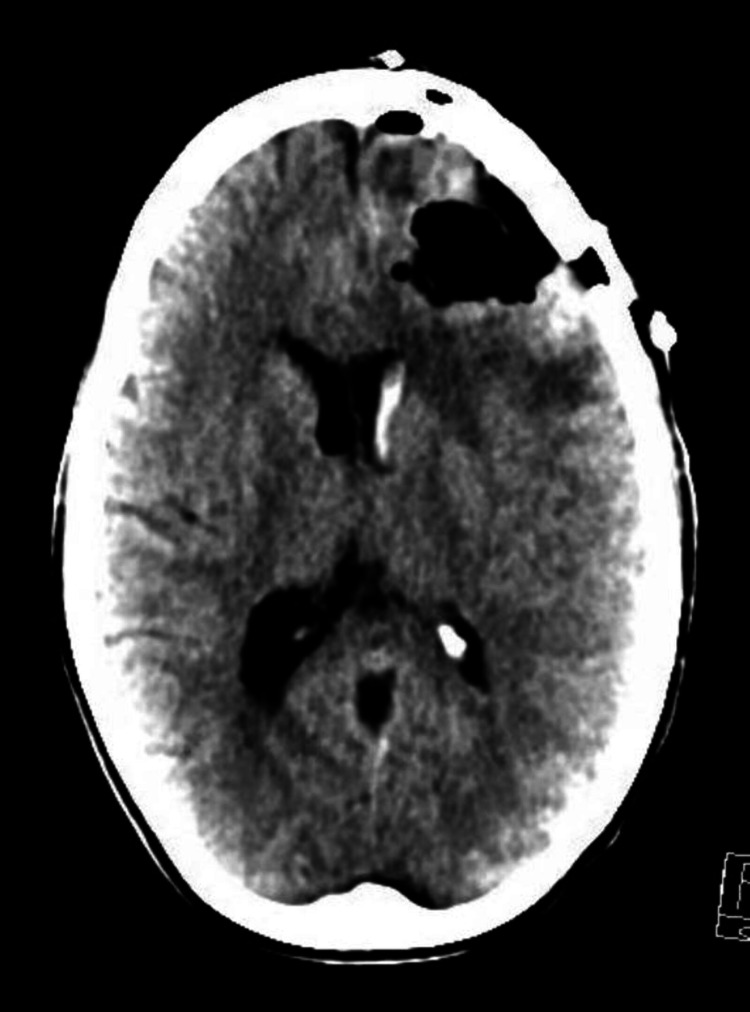
Noncontrast head CT in axial plane post surgical decompression showing a left frontal craniotomy defect from resection of left frontal hemorrhagic mass. There is postoperative hemorrhage and pneumocephalus within the surgical bed. Mass effect is significantly improved with resolution of left-to-right midline shift.

A 56-year-old previously healthy woman without an established primary care physician (PCP) was brought to the emergency department by paramedics for unresponsiveness of unknown duration. In the emergency department, initial vital signs were within normal limits, besides a Glasgow Coma Scale score of six. Physical examination revealed abdominal distension with a large firm mass located in the right upper and lower quadrants. Myoclonic jerks, tongue injury, drooling and evidence of urinary or fecal incontinence were not observed. Laboratory data, including complete blood count, comprehensive metabolic panel, and urine toxicology, were unremarkable. Computed tomography (CT) scan of the brain without contrast showed a large left frontal lobe mass measuring 4.5 x 5.2 cm with acute intraparenchymal hemorrhage, edema, and mass effect (Figure 1). Further evaluation, including CT of the chest, abdomen, and pelvis, revealed a 20.1 x 15.6 x 23.7 cm right retroperitoneal mass originating from the right kidney, suspicious of a renal malignancy and tumor thrombus extending into the IVC (Figure 2). The patient underwent an emergent left full craniotomy to remove the tumor and cerebral hemorrhage (Figure 3). The specimen was sent for pathology with histopathological findings compatible with a metastatic RCC. She was subsequently admitted to the intensive care unit for close monitoring.

The patient was started on dexamethasone to reduce tumor-associated vasogenic cerebral edema and levetiracetam for seizure prophylaxis. The following day, she was alert, awake, oriented, and following commands. The patient later commented that she felt fine before passing out and denied fever, unintentional weight loss, fatigue, headache, dizziness, visual disturbance, abdominal pain, nausea, vomiting, change in bowel habits, hematuria, or seizure activities. Past medical history was significant for fatty liver disease and leiomyoma of the uterus. She denied personal history or known family history of malignancy. She was not taking any medications at home and denied using tobacco, alcohol, or recreational drugs. The patient’s mental status significantly improved over the hospital course, and she was discharged on dexamethasone for 10 days along with levetiracetam. She was cleared by neurosurgery and urology for discharge. She elected to follow-up with urology as an outpatient for cytoreductive nephrectomy evaluation and with oncology for bone scan for completion of staging.

## Discussion

RCC accounts for approximately 2%-3% of all adult malignant neoplasms and 80%-85% of primary renal tumors [3]. Brain metastasis occurs in approximately 8% of patients with RCC [5]. The main symptoms of RCC are flank pain, gross hematuria, and a palpable abdominal mass as the “classic triad”. Additionally, any nonreducing right-sided varicocele with bilateral lower extremity edema can also be seen in patients with advanced disease via the occlusion of the right testicular venous system that drains to the IVC [6]. RCC can also cause paraneoplastic syndrome resulting in hypercalcemia due to parathyroid hormone (PTH)-like secretion, hypertension due to excessive renin production, polycythemia, and thrombocytosis due to excessive erythropoietin production, and Cushing’s syndrome due to excessive cortisol production. Symptoms such as headache, seizures, altered behavior, and confusion may indicate brain metastasis. In this case report, our patient presented with syncope, likely caused by cerebral hypoperfusion from blood vessel compression in the setting of metastatic brain tumor with acute intraparenchymal hemorrhage and surrounding edema.

In regards to diagnostic imaging, a contrast-enhanced, triple-phase helical CT scan is the preferred imaging study to evaluate renal masses as it can identify pathological features [7]. Imaging the brain is recommended only in symptomatic patients, while patients with asymptomatic central nervous system involvement often have worse overall survival [8]. The treatment of RCC brain metastases is complicated and challenging. Before considering treatment options, pathologic confirmation is required. Immunotherapy and chemotherapy used in other forms of RCC metastases play a limited role in RCC brain metastases [6]. Although available treatment plans may improve overall survival, complete remission is rare [9].

This case highlights the importance of annual physical exams, general health screening, and evaluation by PCPs. It is noted that more than 50% of patients with RCC are asymptomatic and, therefore, diagnosed incidentally [10]. A retrospective study reported that about 33% of patients with primary RCC and small metastatic lesions to the brain were asymptomatic [11], which contradicts the general belief that these conditions are primarily symptomatic. With time, all asymptomatic brain metastases become symptomatic if left untreated. Many reports have shown that RCC grows slowly at approximately 0.06-0.39 cm each year [12]. Because of the slow growth rate, metastasis may occur during the long period over which a tumor becomes enormous. With the proper use of thoracoabdominal imaging and routine labs, asymptomatic RCC can be detected earlier than in the past. A renal tumor with a diameter greater than 20 cm is considered extremely rare. In patients with RCC, the relationship between tumor size and prognosis can be confirmed by the tumor-node-metastasis (TNM) classification. More specifically, the five-year survival rate decreases from about 80%-100% in T1 cases to about 50%-80% in T2 cases [13-14]. In our patient, her condition was categorized as poor risk based on the Memorial Sloan-Kettering Cancer Center (MSKCC) prognostic criteria for RCC. Monitoring for symptoms of RCC and early detection via physical exam and routine labs at PCP’s office may enable prompt diagnosis, staging, treatment, and possible prevention of further disease progression and distant metastasis.

## Conclusions

This case demonstrates the importance of early detection of RCC in preventing distant metastasis. Our patient lacked obvious risk factors for RCC and was asymptomatic until the day she presented with syncope. With the slow growth of renal cell cancer, distant metastasis can occur for an extended time without notice. A delay in diagnosis can lead to catastrophic consequences, as seen in our patient. Although periodic screening for RCC in asymptomatic individuals is not recommended unless they have high-risk factors, annual physical exams and proper use of routine labs at PCP’s office may enable prompt diagnosis of RCC and prevention of further complications.
